# Supplementary material for: Salicylic Acid Perturbs sRNA-Gibberellin Regulatory Network in Immune Response of Potato to Potato virus Y Infection
Source: Front Plant Sci. 2017 Dec 22;8:2192. doi: 10.3389/fpls.2017.02192 (PMC5744193; doi:10.3389/fpls.2017.02192)
Supplement: Supplementary file 14 [file Image1.PDF]

A

	D_M1		D_M2		D_M3		D_P1		D_P2		D_P3	
	red.	non-red.	red.	non-red.	red.	non-red.	red.	non-red.	red.	non-red.	red.	non-red.
reads 18-25 nt	7718168	3117729	6718330	3145401	6049356	2584428	13816579	3418557	7285629	2758998	8929554	3445806
low complexity reads	2503827	275347	1366195	122807	1591290	172366	5193816	355379	1725509	148309	1948651	163204
t/rRNA	561794	56883	153569	21485	348673	37148	2110567	37367	408904	24754	455771	26002
genome matched sRNAs	2866133	1468501	3214236	1588475	2592971	1272424	4016294	1639522	3094686	1372485	3936757	1714886
known miRNAs	188117	205	236562	222	202904	214	376334	229	240501	223	364647	236
novel miRNAs/miRNAs*	7478	149	10263	150	9468	151	13833	165	11032	160	14890	167

B

	NahG_M1		NahG_M2		NahG_M3		NahG_P1		NahG_P2		NahG_P3	
	red.	non-red.	red.	non-red.	red.	non-red.	red.	non-red.	red.	non-red.	red.	non-red.
reads 18-25 nt	3234137	1365027	8541852	2429728	4566636	2089905	5748934	2088219	8899956	3164932	4472255	1959596
low complexity reads	1150618	118669	3618989	210099	1123523	140105	1663503	167471	2128342	268039	997972	129799
t/rRNA	222869	28677	655998	37835	270169	33034	439578	39187	810850	62148	294375	34051
genome matched sRNAs	1191167	671143	2818468	1177527	2005330	1031075	2252675	995921	3727295	1464781	1961316	956190
known miRNAs	111103	190	347146	212	186217	212	262077	215	663702	226	266446	210
novel miRNAs/miRNAs*	4878	127	14558	142	8214	144	10535	154	20868	143	11051	148

C

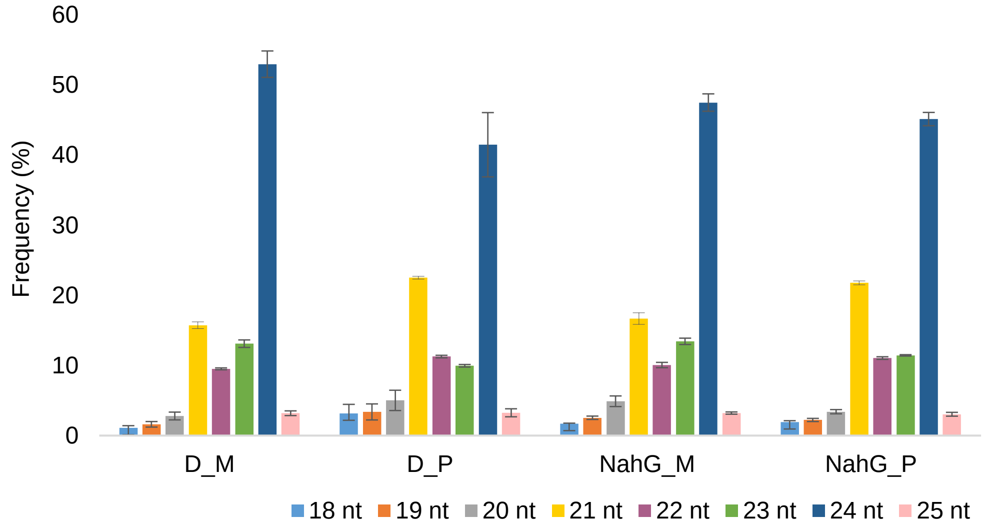

Figure S1. Overview of the sRNA sequencing data set for the analyzed potato samples. The numbers of different categories of sRNA sequences for the control and PVY^{NTN}-infected samples of tolerant (**A**) Désirée and salicylic acid-deficient (**B**) NahG-Désirée, represented as redundant (red.) and non-redundant (non-red.) counts with the read lengths of 18-25 nt, number of low complexity reads that were filtered out, genome matched reads and numbers of known and novel miRNAs and their complementary miRNAs*. (**C**) sRNA sequence length distribution: number of sequences observed at specific sequence length normalized to the total number of sequences from the same treatment. Values are presented as mean ± SE (n=3). The majority of the reads were 20-25-nt in length, of which the 24-nt class was the most abundant (~ 60 %), followed by the 21-nt class. D – Désirée; NahG – NahG-Désirée; M – mock; P – PVY^{NTN}; 1, 2, 3 – biological replicates of individual plants.
