# Supplementary material for: Salicylic Acid Perturbs sRNA-Gibberellin Regulatory Network in Immune Response of Potato to Potato virus Y Infection
Source: Front Plant Sci. 2017 Dec 22;8:2192. doi: 10.3389/fpls.2017.02192 (PMC5744193; doi:10.3389/fpls.2017.02192)
Supplement: Supplementary file 15 [file Image2.PDF]

A Désirée

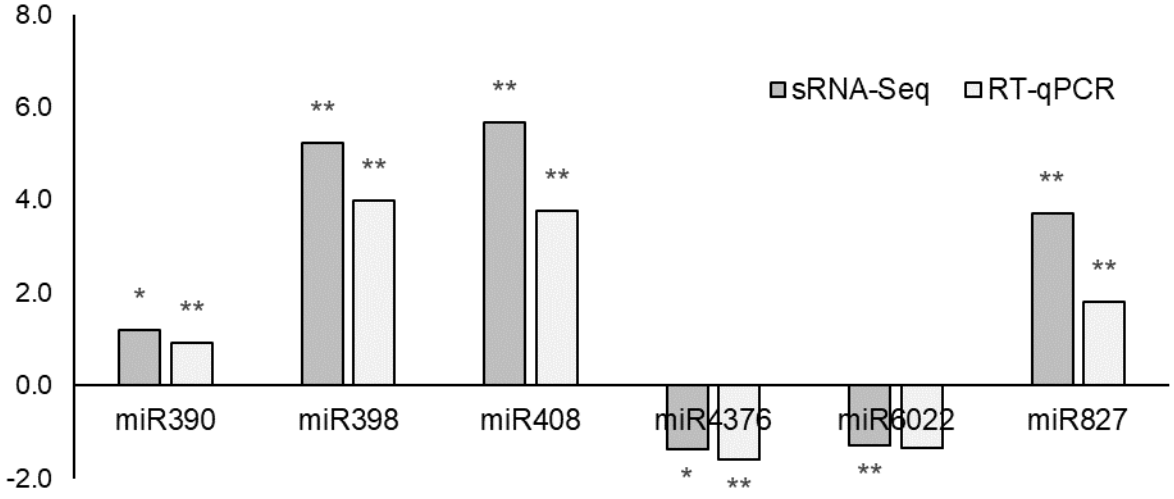

B NahG-Désirée

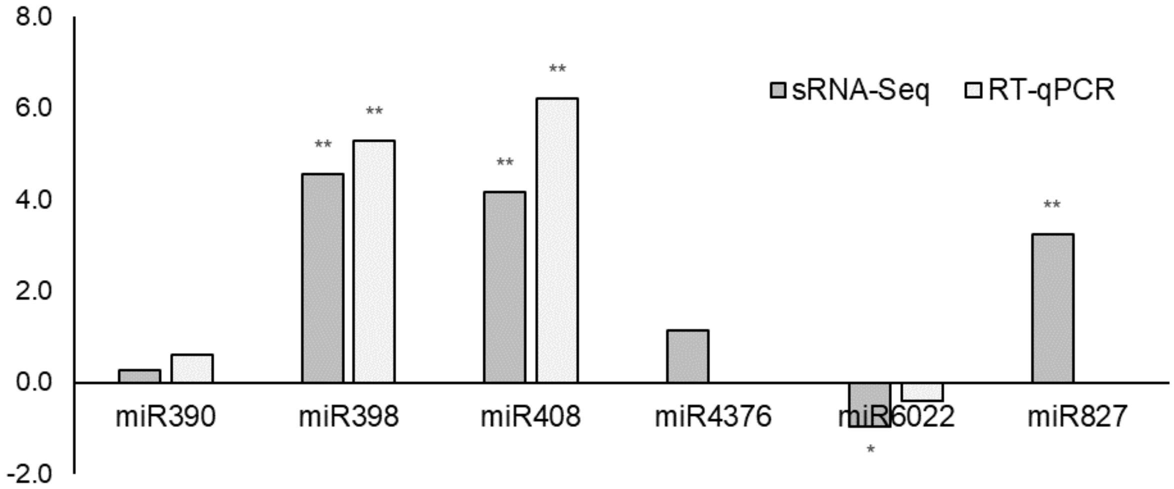

Figure S2. Validation of sRNA-Seq results by stem-loop RT-qPCR. The $\log_2$ ratios of expression of six miRNA in PVY^{NTN}- infected samples versus mock inoculated samples of potato cv. (**A**) Désirée or (**B**) NahG-Désirée as determined by sRNA-Seq (dark gray) or RT-qPCR (light gray). The expressions of miR827 and miR4376 in NahG-Désirée samples were under the limit of quantification. Asterisks indicate a statistically significant changes (**p-value < 0.05; *p-value < 0.1). See **Material and Methods** for details of the experimental procedure.
