# Supplementary material for: Salicylic Acid Perturbs sRNA-Gibberellin Regulatory Network in Immune Response of Potato to Potato virus Y Infection
Source: Front Plant Sci. 2017 Dec 22;8:2192. doi: 10.3389/fpls.2017.02192 (PMC5744193; doi:10.3389/fpls.2017.02192)
Supplement: Supplementary file 16 [file Image3.PDF]

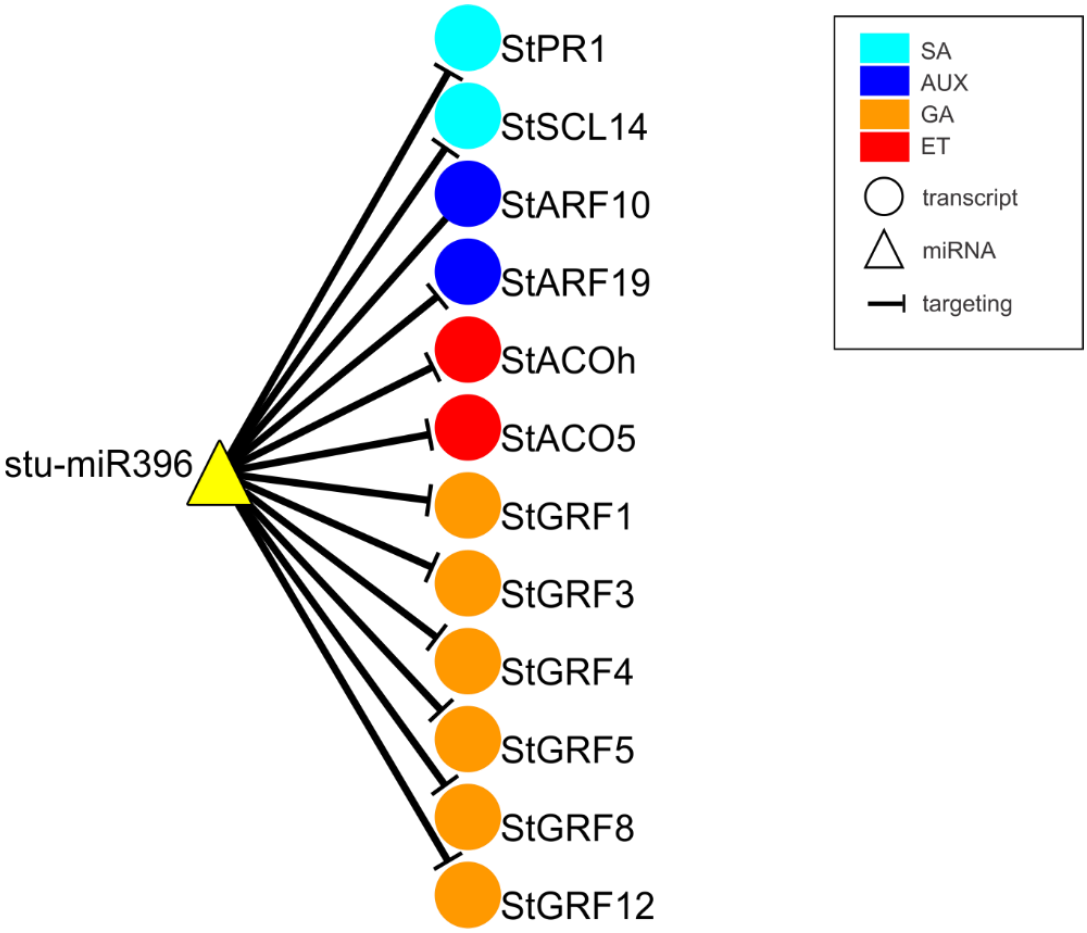

Figure S3. miR396-auxin signaling network. miR396 targets as detected by *in silico* prediction and degradome sequencing. miR396 exhibited diverse target repertoire from already well known conserved targets such as *GRF*s, to transcripts coding for GRAS protein SCARECROW-like 14 (StSCL14), ACOs involved in the ethylene biosynthesis, ARFs involved in auxin signaling and even pathogenesis-related protein 1 (PR1). Thus, besides their important developmental role, miR396 family is also shown to be involved in the control of phytohormone- and defense-related genes. Similarly to the study of Hewezi et al. (2012), in which many miR396-GRF-regulated genes were discovered to be involved in phytohormone signaling pathways. Node shapes represent components: triangle – miRNA; circle – transcripts, while node colors indicate different signaling pathways-related components: turquoise – salicylic acid (SA); blue – auxin (AUX); orange – gibberellin (GA); red – ethylene (ET). Arrows connect miRNA and its targets. Node stu-miR396 represents four miR396 family members stu-miR396-5p, stu-miR396a,b-5p.1, stu-miR396a,b-5p and stu-miR396,a,b-5p.1, node StPR1 represents StPR1.1 and StPR1.2 and node StGRF1 represents StGRF1.1 and StGRF1.2. Gene name symbols are explained in **Table S2**.
