# Supplementary material for: Salicylic Acid Perturbs sRNA-Gibberellin Regulatory Network in Immune Response of Potato to Potato virus Y Infection
Source: Front Plant Sci. 2017 Dec 22;8:2192. doi: 10.3389/fpls.2017.02192 (PMC5744193; doi:10.3389/fpls.2017.02192)
Supplement: Supplementary file 17 [file Image4.PDF]

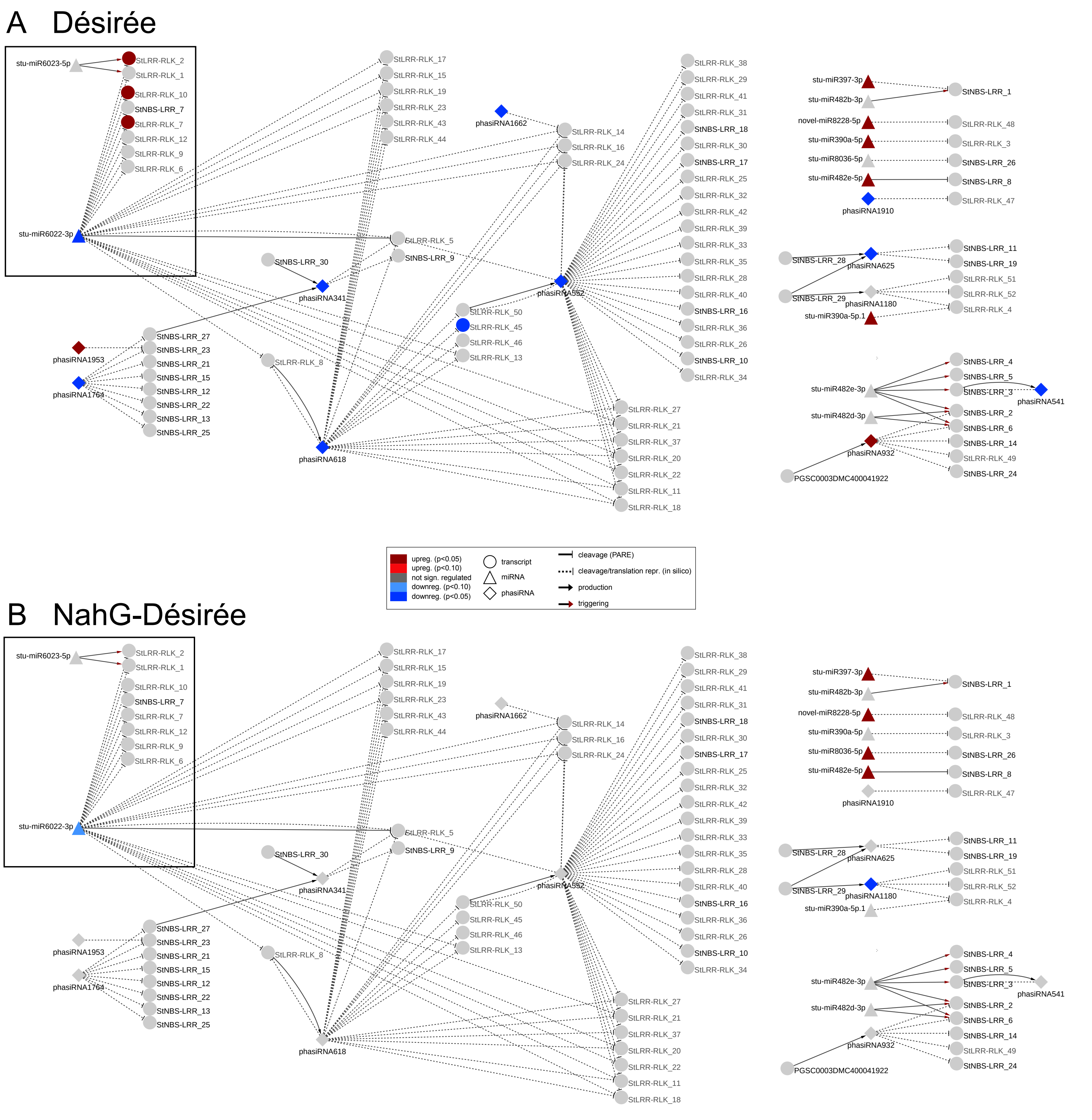
A Désirée
stu-miR6023-5p
StLRR-RLK_2
StLRR-RLK_1
StLRR-RLK_10
StNBS-LRR_7
StLRR-RLK_7
StLRR-RLK_12
StLRR-RLK_9
StLRR-RLK_6
stu-miR6022-3p
StLRR-RLK_17
StLRR-RLK_15
StLRR-RLK_19
StLRR-RLK_23
StLRR-RLK_43
StLRR-RLK_44
phasiRNA1662
StLRR-RLK_14
StLRR-RLK_16
StLRR-RLK_24
StLRR-RLK_38
StLRR-RLK_29
StLRR-RLK_41
StLRR-RLK_31
StNBS-LRR_18
StLRR-RLK_30
StNBS-LRR_17
StLRR-RLK_25
StLRR-RLK_32
StLRR-RLK_42
StLRR-RLK_39
StLRR-RLK_33
StLRR-RLK_35
StLRR-RLK_28
StLRR-RLK_40
StNBS-LRR_16
StLRR-RLK_36
StLRR-RLK_26
StNBS-LRR_10
StLRR-RLK_34
stu-miR397-3p
stu-miR482b-3p
StNBS-LRR_1
novel-miR8228-5p
StLRR-RLK_48
stu-miR390a-5p
StLRR-RLK_3
stu-miR8036-5p
StNBS-LRR_26
stu-miR482e-5p
StNBS-LRR_8
phasiRNA1910
StLRR-RLK_47
StLRR-RLK_5
StNBS-LRR_9
StNBS-LRR_30
phasiRNA341
phasiRNA552
StLRR-RLK_50
StLRR-RLK_45
StLRR-RLK_46
StLRR-RLK_13
StNBS-LRR_28
phasiRNA625
StNBS-LRR_29
phasiRNA1180
stu-miR390a-5p.1
StNBS-LRR_11
StNBS-LRR_19
StLRR-RLK_51
StLRR-RLK_52
StLRR-RLK_4
phasiRNA1953
phasiRNA1764
StNBS-LRR_27
StNBS-LRR_23
StNBS-LRR_21
StNBS-LRR_15
StNBS-LRR_12
StNBS-LRR_22
StNBS-LRR_13
StNBS-LRR_25
StLRR-RLK_8
phasiRNA618
StLRR-RLK_27
StLRR-RLK_21
StLRR-RLK_37
StLRR-RLK_20
StLRR-RLK_22
StLRR-RLK_11
StLRR-RLK_18
stu-miR482e-3p
stu-miR482d-3p
StNBS-LRR_4
StNBS-LRR_5
StNBS-LRR_3
phasiRNA541
StNBS-LRR_2
StNBS-LRR_6
StNBS-LRR_14
StLRR-RLK_49
StNBS-LRR_24
phasiRNA932
PGSC0003DMC400041922
upreg. (p<0.05)
upreg. (p<0.10)
not sign. regulated
downreg. (p<0.10)
downreg. (p<0.05)
transcript
miRNA
phasiRNA
cleavage (PARE)
cleavage/translation repr. (in silico)
production
triggering
B NahG-Désirée
stu-miR6023-5p
StLRR-RLK_2
StLRR-RLK_1
StLRR-RLK_10
StNBS-LRR_7
StLRR-RLK_7
StLRR-RLK_12
StLRR-RLK_9
StLRR-RLK_6
stu-miR6022-3p
StLRR-RLK_17
StLRR-RLK_15
StLRR-RLK_19
StLRR-RLK_23
StLRR-RLK_43
StLRR-RLK_44
phasiRNA1662
StLRR-RLK_14
StLRR-RLK_16
StLRR-RLK_24
StLRR-RLK_38
StLRR-RLK_29
StLRR-RLK_41
StLRR-RLK_31
StNBS-LRR_18
StLRR-RLK_30
StNBS-LRR_17
StLRR-RLK_25
StLRR-RLK_32
StLRR-RLK_42
StLRR-RLK_39
StLRR-RLK_33
StLRR-RLK_35
StLRR-RLK_28
StLRR-RLK_40
StNBS-LRR_16
StLRR-RLK_36
StLRR-RLK_26
StNBS-LRR_10
StLRR-RLK_34
stu-miR397-3p
stu-miR482b-3p
StNBS-LRR_1
novel-miR8228-5p
StLRR-RLK_48
stu-miR390a-5p
StLRR-RLK_3
stu-miR8036-5p
StNBS-LRR_26
stu-miR482e-5p
StNBS-LRR_8
phasiRNA1910
StLRR-RLK_47
StLRR-RLK_5
StNBS-LRR_9
StNBS-LRR_30
phasiRNA341
phasiRNA552
StLRR-RLK_50
StLRR-RLK_45
StLRR-RLK_46
StLRR-RLK_13
StNBS-LRR_28
phasiRNA625
StNBS-LRR_29
phasiRNA1180
stu-miR390a-5p.1
StNBS-LRR_11
StNBS-LRR_19
StLRR-RLK_51
StLRR-RLK_52
StLRR-RLK_4
phasiRNA1953
phasiRNA1764
StNBS-LRR_27
StNBS-LRR_23
StNBS-LRR_21
StNBS-LRR_15
StNBS-LRR_12
StNBS-LRR_22
StNBS-LRR_13
StNBS-LRR_25
StLRR-RLK_8
phasiRNA618
StLRR-RLK_27
StLRR-RLK_21
StLRR-RLK_37
StLRR-RLK_20
StLRR-RLK_22
StLRR-RLK_11
StLRR-RLK_18
stu-miR482e-3p
stu-miR482d-3p
StNBS-LRR_4
StNBS-LRR_5
StNBS-LRR_3
phasiRNA541
StNBS-LRR_2
StNBS-LRR_6
StNBS-LRR_14
StLRR-RLK_49
StNBS-LRR_24
phasiRNA932
PGSC0003DMC400041922

Figure S4. Regulatory network of sRNAs and *R*-genes. *NBS-LRRs* and *LRR-RPK*s targets of differentially expressed sRNAs in (**A**) Désirée and (**B**) NahG-Désirée plants at the onset of viral replication. Node shapes represent different components: triangle – miRNA; diamond – phasiRNA; circle – transcript and rectangle – metabolite. Node colors indicate expression changes: red – upregulated; blue – downregulated and grey – no significant difference in expression. Blocked solid arrow – cleavage observed by degradome-sequencing, blocked dashed-line arrow – *in silico* predicted cleavage, dashed-line arrow with circle – *in silico* predicted translational repression. Black solid line with red arrow connects miRNA trigger and their *PHAS* loci. Black solid line with black arrow connects *PHAS* locus and its producing phasiRNAs. Black rectangle indicates miR6022-LRR-RLKs interaction. For more details of interactions between sRNA-target see **Dataset S6 and S7**.
