# Supplementary material for: Salicylic Acid Perturbs sRNA-Gibberellin Regulatory Network in Immune Response of Potato to Potato virus Y Infection
Source: Front Plant Sci. 2017 Dec 22;8:2192. doi: 10.3389/fpls.2017.02192 (PMC5744193; doi:10.3389/fpls.2017.02192)
Supplement: Supplementary file 18 [file Image5.PDF]

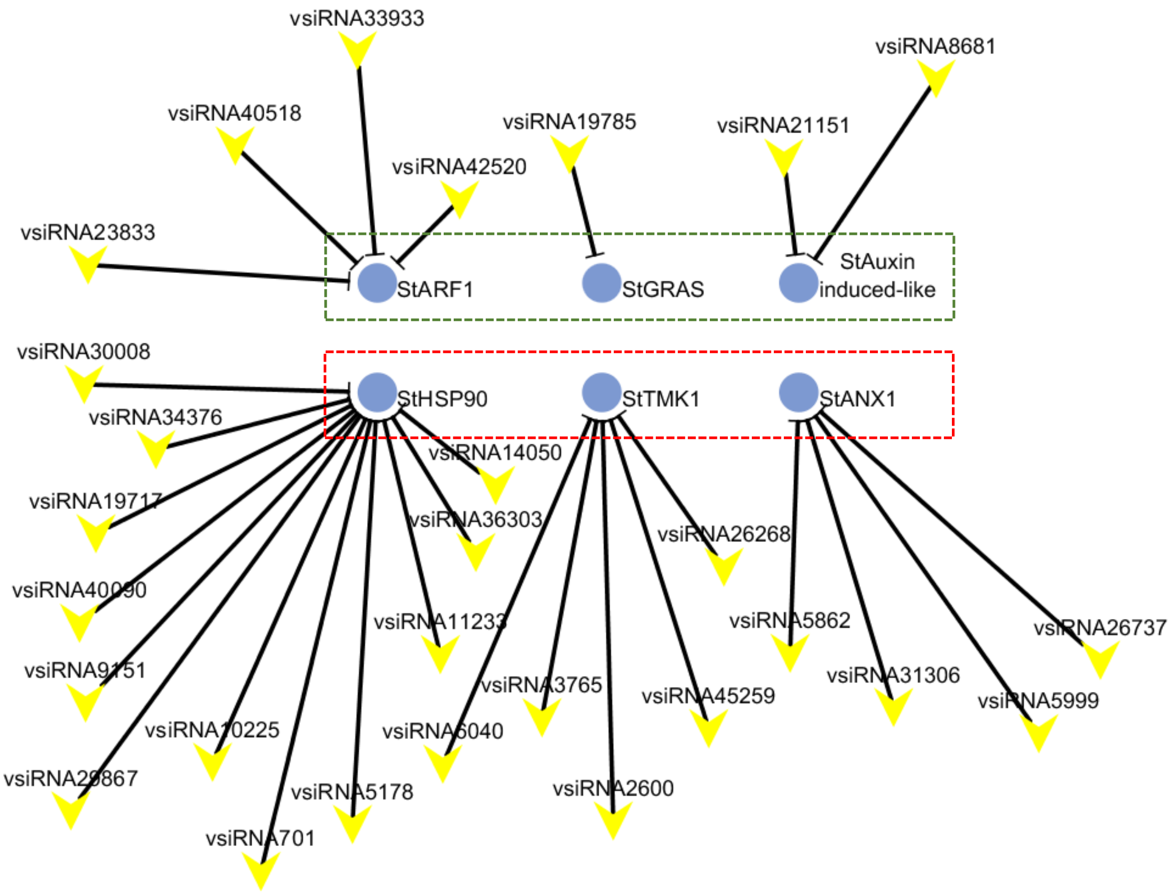

Figure S5. vsiRNAs guide the cleavage of host transcripts involved in immune, growth and developmental signaling. Network shows a set of vsiRNAs and their targets, identified by degradome sequencing. Only a subset of targets with reduced expression level following PVY^{NTN} infection in the tolerant plants of Désirée are shown (FDR-adjusted p-value < 0.05; Stare et al. 2015). Node shapes represent different components: arrowhead – vsiRNA, circle – target transcripts. Green rectangle indicates growth and development related transcripts, red rectangle indicates targets involved in immune signaling. StHSP90 – Heat shock protein 90, StTMK1 – Leucine rich receptor like kinase TMK1, StANX1 – Leucine rich receptor like kinase ANX1, StARF1 – Auxin response factor 1, StGRAS –transcription factor GRAS, StAuxin induced-like – Auxin induced-like protein F25G13.70.
