# Supplementary material for: Salicylic Acid Perturbs sRNA-Gibberellin Regulatory Network in Immune Response of Potato to Potato virus Y Infection
Source: Front Plant Sci. 2017 Dec 22;8:2192. doi: 10.3389/fpls.2017.02192 (PMC5744193; doi:10.3389/fpls.2017.02192)
Supplement: Supplementary file 19 [file Table1.PDF]

Table S1. RT-qPCR assays for selected miRNAs used for sRNA-Seq results validation.

TaqMan MicroRNA Assays (Thermo Fisher Scientific), ordered according to the sRNA-Seq sequence of the selected miRNAs together with their IDs, mature miRNA sequence and efficiency of amplification.

Assay name	Assay ID (Thermo Fisher Scientific)	Mature miRNA sequence	Efficiency of amplification (%)
gma-miR390b	007109_mat	AAGCTCAGGAGGGATAGCACC	106.5
stu-miR398a-5p	469260_mat	GGGTTGATTTGAGAACATATG	91.6
stu-miR408b-5p	468849_mat	ACGGGGACGAGACAGAGCATG	104.2
sly-miR4376	473102_mat	ACGCAGGAGAGATGATGCTGGA	104.6
bna-miR167a	006933_mat	TGAAGCTGCCAGCATGATCTAA	98.3
stu-miR6022	475531_mat	TGGAAGGGAGAATATCCAGGA	100.7
stu-miR827-5p	468282_mat	TTTGTTGATGGTCATCTATTC	105.6
