# Supplementary material for: Salicylic Acid Perturbs sRNA-Gibberellin Regulatory Network in Immune Response of Potato to Potato virus Y Infection
Source: Front Plant Sci. 2017 Dec 22;8:2192. doi: 10.3389/fpls.2017.02192 (PMC5744193; doi:10.3389/fpls.2017.02192)
Supplement: Supplementary file 20 [file Table2.PDF]

Table S2. List of genes referred to in the manuscript together with their descriptions and corresponding IDs.

Gene symbols and potato genes names, their full descriptions and corresponding potato StNIB_v1 identifiers (IDs; Ramšak et al., 2014), together with potato IDs of paralogous genes (potato gene and unigene sequences StNIB_v1), used in the manuscript, are shown. Potato genes were named according to the *Arabidopsis thaliana* orthologs, obtained from GoMapMan (www.gomapman.org; Ramšak et al., 2014).

Gene symbol	Potato gene name	StNIB_v1 ID	Full description	IDs of all paralogs	*Arabidopsis thaliana* ortholog ID
**ACD1**	**StACD1**	**Sotub03g022940**	1-aminocyclopropane-1-carboxylic acid deaminase 1	Sotub03g022940	AT1G48420
**ACO5**	**StACO5**	**Sotub02g024300**	1-aminocyclopropane-1-carboxylate oxidase 5	MICRO.2939.C2; MICRO.2939.C3; TC197510; TC213446	AT1G05010
**ACOh**	**StACOh**	**Sotub09g026280**	1-aminocyclopropane-1-carboxylate oxidase homolog	Sotub09g026280; PGSC0003DMC400030140	AT1G03400; AT1G03410
**AFB**	**StAFB1.1**	**Sotub06g008630**	Auxin F-box protein 1	Sotub06g008630; TC197735; MICRO.9052.C2; MICRO.9052.C1; PGSC0003DMC400009476	AT1G12820; AT2G39940; AT3G26810; AT3G62980; AT4G03190; AT4G24390; AT5G49980
**AFB**	**StAFB1.2**	**Sotub06g008900**	Auxin F-box protein 1	Sotub06g008900; Sotub06g008780; PGSC0003DMC400036051; MICRO.13137.C1; TC219660; TC215657; Sotub06g008800	AT1G12820; AT2G39940; AT3G26810; AT3G62980; AT4G03190; AT4G24390; AT5G49980
**AFB**	**StAFB1.3**	**Sotub06g034990**	Auxin F-box protein 1	Sotub06g034990; PGSC0003DMC400041556	AT1G12820; AT3G26810; AT3G62980; AT4G03190
**AFB**	**StAFB2**	**Sotub06g008730**	Auxin signaling F-box 2	Sotub06g008730; PGSC0003DMC400038062; PGSC0003DMC400038063	AT1G12820; AT3G26810; AT3G62980; AT4G03190
**AFB**	**StAFB3**	**Sotub06g008920**	Auxin signaling F-box 3	PGSC0003DMC400064891; MICRO.16535.C1; TC215350; EG013617; SDBN005N07u.scf	AT1G12820; AT2G39940; AT3G26810; AT3G62980; AT4G03190; AT4G24390; AT5G49980
**AFB**	**StAFB5**	**Sotub02g022180**	Auxin F-box protein 5	Sotub02g022180; PGSC0003DMC400038842; MICRO.15100.C1; TC196340	AT1G12820; AT3G26810; AT3G62980; AT4G03190
**ANX1**	**StANX1**	**Sotub09g014230**	Leucine-rich repeat receptor-like kinase	Sotub09g014230	AT3G51550
**AP2**	**StAP2**	**Sotub10g024610**	APETALA2	Sotub10g024610; PGSC0003DMC400021291; Sotub04g013820; PGSC0003DMC400021292; PGSC0003DMC400021293; PGSC0003DMC400019509; MICRO.5083.C3; TC217078; PGSC0003DMC400019512; Sotub02g031630; PGSC0003DMC400019514; PGSC0003DMC400007140; MICRO.5083.C1; PGSC0003DMC400019513; TC208874; TC211656; CK249226; MICRO.7449.C1	AT4G36920
**ARF1**	**StARF1.1**	**Sotub01g040050**	Auxin response Factor 1	Sotub01g040050	AT1G59750
**ARF1**	**StARF1.2**	**Sotub01g038660**	Auxin response Factor 1	Sotub01g038660	AT3G62290
**ARF2**	**StARF2**	**Sotub03g031730**	Auxin response factor 2	Sotub03g031730	AT5G62000
**ARF10**	**StARF10**	**Sotub11g026490**	Auxin response factor 10	Sotub11g014110; PGSC0003DMC400048339	AT2G28350; AT4G30080
**ARF19**	**StARF19**	**Sotub07g008830**	Auxin response factor 19	Sotub05g021800; PGSC0003DMC400024204	AT1G19220; AT5G20730
**DELLA**	**StDELLA**	**Sotub02g037540**	DELLA protein	Sotub11g008520; PGSC0003DMC400027527	AT1G14920; AT2G01570; AT3G03450
**DWF4**	**StDWF4**	**Sotub02g028500**	Dwarf4	Sotub02g028500	AT3G50660
**EIN4**	**StEIN4**	**Sotub11g005240**	Ethylene insensitive 4	Sotub11g005240	AT3G04580
**ERF2a**	**StERF2a**	**Sotub01g009690**	Ethylene responsive transcription factor 2a	PGSC0003DMG400046911;Sotub01g009590.1.1; Sotub01g009600.1.1;Sotub01g009610.1.1;Sotub01g009640.1.1;Sotub01g009660.1.1;Sotub01g009670.1.1;Sotub01g009690.1.1	AT1G03800; AT1G04370
**F25G13.70**	**StF25G13.70**	**Sotub05g018740**	Auxin induced-like protein	Sotub05g018740	AT3G25290; AT4G12980; AT4G17280; AT5G35735; AT5G47530
**GA1**	**StGA1**	**Sotub06g034690**	GA requiring 1	Sotub06g034690	AT4G02780
**GA20ox**	**StGA20ox**	**Sotub10g011620**	Gibberellin 20-oxidase	Sotub10g011620; PGSC0003DMC400061378	AT4G25420AT5G51810
**GA20ox**	**StGA20ox1**	**Sotub03g007160**	Gibberellin 20-oxidase-1	Sotub03g007160; Sotub06g009210; PGSC0003DMC400041949	AT4G25420
**GA20ox**	**StGA20ox3**	**Sotub11g029030**	Gibberellin 20-oxidase 3	Sotub11g029030	AT4G25420AT5G51810
**GA20ox**	**StGA20ox4**	**Sotub01g031210**	Gibberellin 20-oxidase 4	Sotub01g031210; MICRO.15941.C1; PGSC0003DMC400000034; PGSC0003DMC400000035	AT4G25420AT5G51810
**GA3ox**	**StGA3ox**	**Sotub06g023360**	Gibberellin 3-oxidase	Sotub06g023360; PGSC0003DMC400028884	AT1G15550; AT1G80340; AT4G21690

**GRF1**	**StGRF1.1**	**Sotub02g030550**	Growth-regulating factor 1	BF186907; MICRO.328.C1; Sotub04g030450; PGSC0003DMC400008794; PGSC0003DMC400037092; Sotub02g030550; TC211003	AT2G22840; AT4G37740
**GRF1**	**StGRF1.2**	**Sotub07g014780**	Growth-regulating factor 1	Sotub07g014780; MICRO.3833.C1; TC217359; PGSC0003DMC400023044	AT2G06200
**GRF3**	**StGRF3**	**Sotub08g008530**	Growth-regulating factor 3	Sotub08g008530; cSTB21A15TH; PGSC0003DMC400032221; Sotub08g021020; PGSC0003DMC400053791; PGSC0003DMC400032220; PGSC0003DMC400032219; PGSC0003DMC400032217	AT2G06200; AT2G22840; AT2G36400; AT2G45480; AT3G13960; AT3G52910; AT4G24150; AT4G37740; AT5G53660
**GRF4**	**StGRF4**	**Sotub03g016800**	Growth-regulating factor 4	Sotub03g016800; PGSC0003DMC400005813	AT5G53660
**GRF5**	**StGRF5**	**Sotub12g028430**	Growth-regulating factor 5	MICRO.4557.C1; PGSC0003DMC400051174	AT2G06200; AT2G22840; AT2G36400; AT2G45480; AT3G13960; AT3G52910; AT4G24150; AT4G37740; AT5G53660
**GRF5**	**StGRF5**	**Sotub12g028430**	Growth-regulating factor 5	MICRO.4557.C1; PGSC0003DMC400051174	AT2G06200; AT2G22840; AT2G36400; AT2G45480; AT3G13960; AT3G52910; AT4G24150; AT4G37740; AT5G53660
**GRF8**	**StGRF8**	**Sotub08g027110**	Growth-regulating factor 8	Sotub08g027110; TC219670; MICRO.1264.C1; PGSC0003DMC400021643	AT4G24150
**GRF12**	**StGRF12**	**Sotub01g030660**	Growth-regulating factor 12	Sotub01g030660; PGSC0003DMC400022619	AT2G45480
**HSP90**	**StHSP90**	**Sotub12g013560**	Heat shock protein 90	Sotub12g013560	AT5G52640; AT5G56000; AT5G56010; AT5G56030
**IAA16**	**StIAA16**	**Sotub01g034780**	Indoleacetic acid-induced protein 16	Sotub01g034780	AT3G04730
**IAR3**	**StIAR3**	**Sotub12g009860**	IAA-amino acid hydrolase IAR3	Sotub12g009860	AT1G51760; AT1G51780
**ILR1**	**StILR1**	**Sotub06g018160**	IAA-amino acid hydrolase ILR1	Sotub06g018160	AT3G02875
**LOX1**	**StLOX1**	**Sotub01g036960**	Lipoxygenase 1	Sotub01g036960	AT1G55020
**LRR-RLK**	**StLRR-RLK**	**Sotub01g006120**	Leucine-rich repeat receptor-like kinase	Sotub01g006120; Sotub01g006170; Sotub01g017420;	AT1G71390; AT1G71400
**MYB33**	**StMYB33**	**Sotub06g030530**	MYB domain protein 33	Sotub06g030530	AT5G06100
**OPR1**	**StOPR1**	**Sotub11g017720**	12-oxophytodienoate (OPDA) reductase	Sotub11g017720	AT1G76680; AT1G76690
**PR1**	**StPR1.1**	**Sotub01g043930**	Pathogenesis-related protein 1	Sotub09g006110	AT1G50060
**PR1**	**StPR1.2**	**Sotub01g043880**	Pathogenesis-related protein 1	TC224536	AT2G14580; AT2G14610
**SAUR45**	**StSAUR45**	**Sotub10g025790**	Small auxin upregulated RNA 45	Sotub10g025790	AT2G36210
**SCL14**	**StSCL14**	**Sotub04g010480**	Scarecrow-like 14	Sotub04g010480; PGSC0003DMC400011269	AT1G07520; AT1G07530; AT2G29060; AT2G37650
**SN1**	**StSN1**	**Sotub04g031180**	Snakin-1	Sotub04g031180	AT2G14900
**SPL11**	**StSPL11**	**Sotub05g016440**	Squamosa promoter-binding-like protein 11	Sotub05g016440	AT5G43270
**TIR1**	**StTIR1**	**Sotub09g022650**	Transport inhibitor response 1	Sotub09g022650; PGSC0003DMC400020096; POADF41TV; PGSC0003DMC400020095; MICRO.1332.C1; TC213418	AT3G62980
**TMK1**	**StTMK1**	**Sotub01g046210**	Receptor-like serine/threonine-protein kinase	Sotub01g046210	AT2G01820; AT1G66150
